# Carbonation Rates of Dry Ca(OH)_2_ Mortars for CO_2_ Capture Applications at Ambient Temperatures

**DOI:** 10.1021/acs.iecr.2c01675

**Published:** 2022-09-27

**Authors:** Yolanda A. Criado, J. Carlos Abanades

**Affiliations:** CSIC-INCAR, C/Francisco Pintado Fe, 26, 33011 Oviedo, Spain

## Abstract

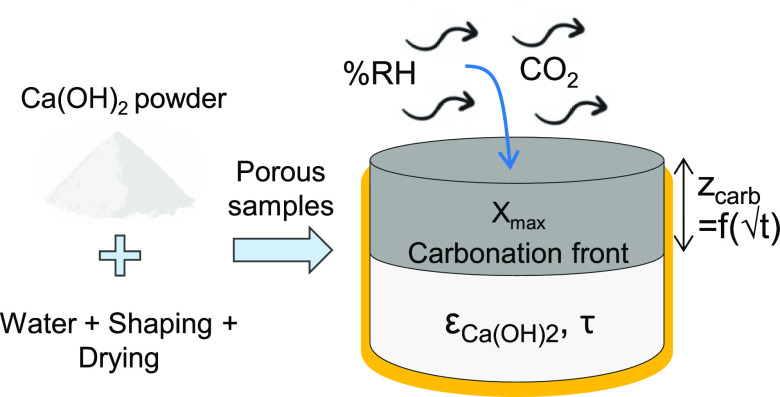

The carbonation rates of porous mortars, pellets, and extruded forms of Ca(OH)_2_ were determined to investigate their suitability as functional materials for direct air capture. Samples of 4–15 mm thickness and porosities between 0.2 and 0.8 were tested by monitoring the progress of the carbonation fronts on time scales from 1 to 500 h. The evolution of such carbonation fronts was found to obey Fick’s diffusion law under all tested conditions. To reach CaCO_3_ conversions higher than 0.6, a relative humidity above 50%, preferably between 80 and 100%, was required when using dry, low-grade slaked lime with a surface area of 18 m^2^/g as CO_2_ sorbent. For modest relative humidities of 50%, higher grades of Ca(OH)_2_ (i.e., with a surface area approaching 40 m^2^/g) still allowed carbonation conversions above 0.8. The results confirm the applicability of these commercial solids for the direct air capture of CO_2_.

## Introduction

The carbonation of Ca(OH)_2_-containing solids under ambient conditions has been studied by a number of researchers investigating the fundamentals of this gas–solid reaction.^1−9^ This type of carbonation has also been investigated because of its importance with regard to mortars^10−12^ and as the basis of a method for direct CO_2_ capture from the atmosphere.^9,13−18^ Regarding the latter, we have recently investigated^19,20^ the possibility of deploying vast volumes of purpose-built passive carbonation infrastructures of porous Ca(OH)_2_ stacked in such a way as to maximize the exposure of active Ca(OH)_2_ surfaces to ambient air. The development of these applications could take advantage of the low specific cost of Ca(OH)_2_ precursor (i.e., limestone) in many locations as well as the near-term availability of technologies for the oxy-calcination of CaCO_3_ for cement production,^21,22^ calcium looping,^23^ and other direct air capture systems using CaO.^24−26^

Most of the works studying the carbonation reaction of Ca(OH)_2_ in powder form with the ambient air suggest that the reaction mechanism under such operational conditions^1,3^ differs from the gas–solid reaction mechanism observed for the carbonation of Ca-based materials under temperatures above 100 °C. Beruto and Botter^1^ investigated the effect of relative humidity in the air to promote faster carbonation of Ca(OH)_2_ powders, reporting extremely slow carbonation rates for the carbonation of Ca(OH)_2_ under dry conditions. Following these observations, a reaction mechanism was proposed based on the adsorption of liquid water on the internal surface of the particles to promote the carbonation reaction at low temperatures. This reaction mechanism was also proposed by Dheilly et al.^2^ and Shih et al.^3^ when studying the reaction at temperatures below 90 °C and CO_2_ concentrations from 0.03 %v up to 12 %v and has been observed as well for the carbonation of other Ca-based materials in the cement chemistry.^10−12^ In addition to this, several authors^3,5,7^ observed linear relations between the CO_2_ uptake and the Ca(OH)_2_ material surface area^3,5^ as well as improved reaction rates as the surface area increases.^7^ More recently, Erans et al.^9^ have investigated this reaction for direct air capture applications, requiring times up to 500–1000 h to carbonate 5 mm deep layers of CaO and Ca(OH)_2_ powders. These reaction times are in agreement with those reported in the state-of-the-art carbonation of large concrete structures, the carbonation rates of such structures being controlled by pore diffusion.^27^

In this work, we experimentally investigate the carbonation process of Ca(OH)_2_ porous solids with potential applicability in passive carbonation schemes. To this end, pelletized samples of Ca(OH)_2_ powder, dry mortars of Ca(OH)_2_ and water, and extruded shapes of the same mortars (i.e., brick-shape) are tested under different conditions to monitor the progress of the carbonation fronts over time and develop models that can be used for the design of large-scale systems for direct CO_2_ capture with Ca(OH)_2_.

## Experimental Section

### Materials

Ca(OH)_2_ commercial powders were used to prepare all samples: dry slaked lime, used for construction applications, and a high-grade Ca(OH)_2_, used for environmental applications. These powders have an average particle size of approximately 5–6 μm (measured by a Beckman–Coulter LS 13320 laser diffraction analyzer using ethanol as a dispersant agent), a true density of approximately 2.2 g/cm^3^ (measured by helium pycnometry with an AccuPyc II-1340, Micromeritics Corp.), and a main impurity of CaCO_3_ (between 3 and 8% wt, as measured by LECO CS130). Their surface areas and pore diameters are, however, very different: 18.2 and 39.3 m^2^/g and 223 and 68 nm, respectively, for dry slaked lime and high-grade Ca(OH)_2_, measured by Ar adsorption at −196 °C with an ASAP 2020 instrument, Micromeritics Corp., and applying the BET equation and mercury pycnometry with Autopore IV 9500 instruments, Micromeritics Corp.

### Preparation of Ca(OH)_2_ Porous Samples

Three different preparation methods were used to obtain porous solid samples with different initial porosities and characteristic lengths: the pelletization of dry powder (under a range of different pressures), the preparation of mortars with water that were then dried in molds to obtain the desired shapes, and the extrusion of mortars to obtain brick-like shapes.

Because the bulk open porosity of solids is known to be a major variable during the carbonation of Ca materials in ambient air,^10,16,27,28^ a wide range of porosities, between 0.2 and 0.8, were tested. To obtain highly porous samples (i.e., with porosities ε_Ca(OH)_2__ between 0.6 and 0.8), dry Ca(OH)_2_ powder was placed in cylindrical plastic vessels 1.5 cm in diameter and height, with the top open as the only contacting surface. Then, the powder was compressed using a dynamometer (SHIMPO FGE-100X) by applying forces of up to 200 N. The porosities of the resulting compacted beds were determined by comparing the Ca(OH)_2_ true density with that calculated from the weight and volume occupied by the compressed powder inside the vessels. A similar dry preparation method was used to obtain Ca(OH)_2_ pellets with porosities between 0.2 and 0.4. The Ca(OH)_2_ powder was shaped in a TDP-1.5 single-punch tablet manual press fitted with a 550 W engine that supplied a maximum pressure of 15 kN, producing cylindrical pellets 6 mm in diameter and approximately 4 mm in height.

Wet mortars were prepared by mixing Ca(OH)_2_ powder with approximately 40% wt distilled water, allowing the shaping of the mortar in molds of dimensions comparable to the dry samples prepared above. The conformed mortar was then dried in an oven at 110 °C under N_2_. After drying, the pellets were weighed in a precision balance and their final dimensions were measured to calculate their initial porosity. From this method, samples with ε_Ca(OH)_2__ values between 0.4 and 0.6 were obtained.

**Figure 1:**
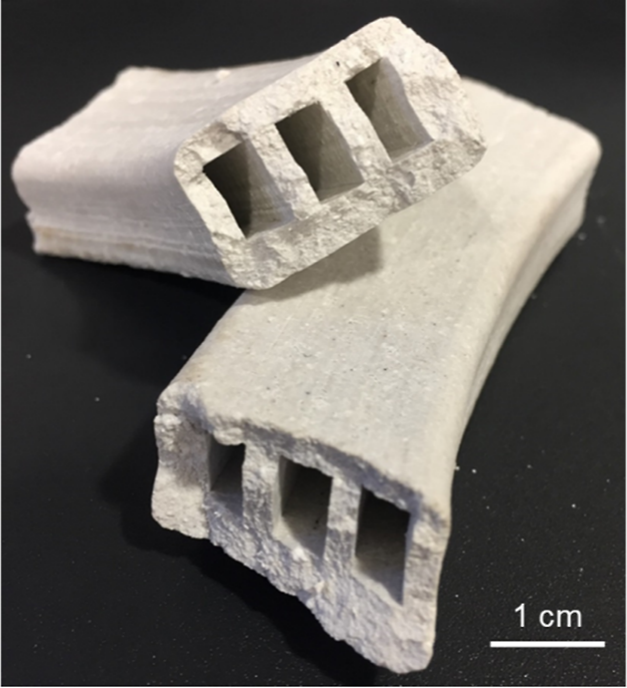
Photograph of a brick-shaped Ca(OH)_2_ sample obtained from wet mortar extruded with the DEX LAB equipment and then dried.

Finally, wet mortars of a similar likeness to those above were extruded in a laboratory-scale extruder machine designed and manufactured by Talleres Felipe Verdés S.A.^29^ The equipment (model DEX LAB) is based on a helix 80 mm in diameter capable of providing up to 7.5 kW and 80 bar for extrusion. The extrusion velocity can be adjusted to between 5 and 15 rpm, allowing for a maximum production of 500 kg/h. From wet mortar (with approximately 32% wt humidity), brick-like shapes were produced and then dried in an oven at 110 °C overnight. Figure 1 shows an image of one of the obtained extruded forms. The motivation for investigating these brick-shaped Ca(OH)_2_ materials is the possible use of carbonated bricks as construction materials. However, for the scope of the investigation described in this work, these extruded brick shapes are not too different from the handmade mortars of similar dimensions and porosities when tested under ambient conditions, as will be shown below.

### Carbonation Tests

Carbonation experiments were carried out on the samples in three setups: by simply allowing their carbonation to proceed in a test room, by arranging the samples in a sealed glass container surrounded by a thermostatic bath to ensure a homogeneous temperature and fluxed by air containing CO_2_ with a high relative humidity, and by using a thermogravimetric analyzer (TGA) fully described elsewhere^30^ to test the carbonation of the powdered samples of parent solids and pellet samples of less than 100 mg.

**Figure 2:**
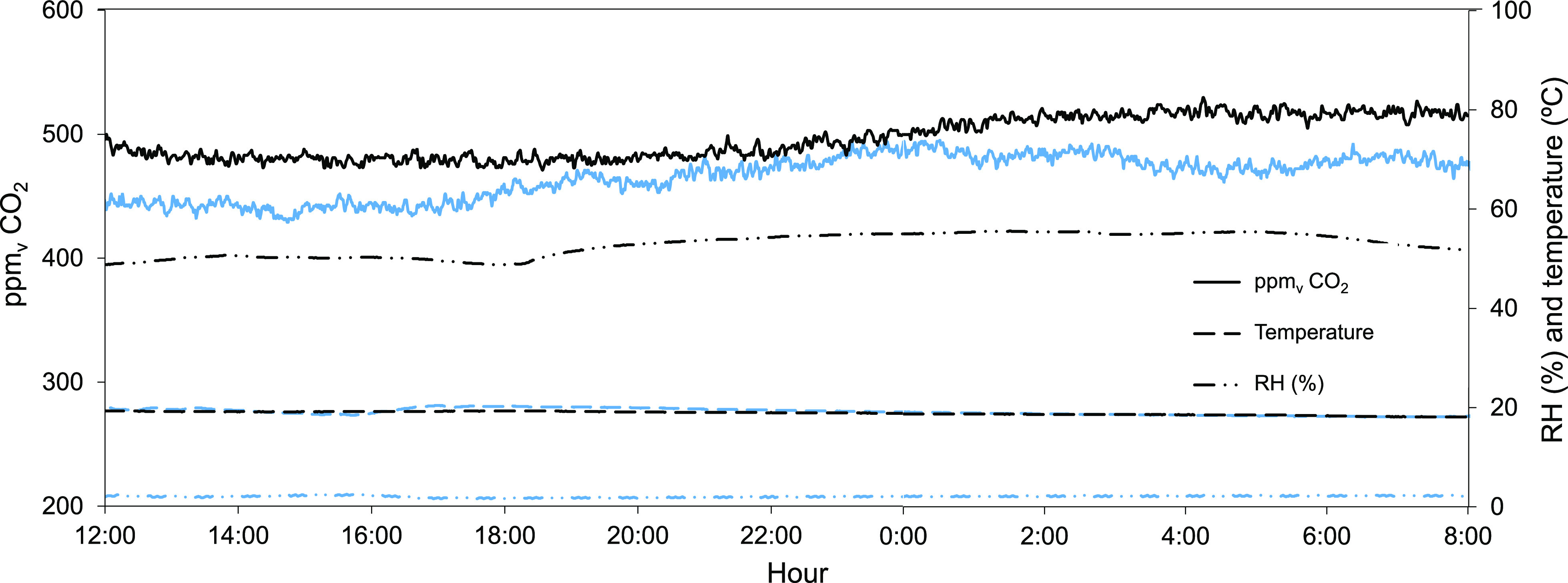
Illustrative variation over time of the CO_2_ concentration (in ppm_v_), relative humidity (RH) and temperature in the test room (black lines), and compressed air (blue lines).

The tests under ambient room conditions typically lasted 500 h. Such a long carbonation time facilitates the frequent weighing of the samples and the use of destructive analysis by arranging the carbonation of several samples in parallel. The CO_2_ concentration in the test room was 500 ± 25 ppm_v_ CO_2_, the relative humidity (RH) was 52 ± 3%, and the temperature was 19 ± 2 °C, as shown in Figure 2, which were measured by analyzing the ambient conditions using a PCE-AQD 10 Air Quality Data Logger device (for measuring over the ranges of 0 to 4000 ppm_v_ CO_2_, 10–90% RH and 0–50 °C). A fan was located in front of the samples to ensure a velocity of approximately 0.5 m/s in their proximity, thus allowing differential conditions with respect to the ambient air.

In the setup designed for carbonation under conditions of high RH, the samples were placed in a sealed glass container situated in a thermostatic bath (JP Selecta Unitronic 27 L for temperatures of 5–99.9 °C). The system was heated at controlled temperatures between 20 and 80 °C and flushed by a flow of about 200 L/h of compressed air. As shown in Figure 2, the concentration of CO_2_ in the compressed air was 450 ± 35 ppm_v_ CO_2_, and the RH was below 2%. This air flow was then saturated with humidity by bubbling it in a distilled water glass placed in the thermostatic bath. To avoid condensation and heat losses, the top of the glass container was heated using a heating cord, and the whole system was covered with insulated material. Additionally, in this setup, some accelerated carbonation tests (typically less than 24 h to reach maximum conversions) were carried out with CO_2_ concentrations 2 orders of magnitude larger than those in ambient air (8 and 12 %v CO_2_), as will be discussed below.

When testing the solid samples either in the glass container or in the testing room, their lateral and bottom surfaces were covered, thus allowing the carbonation process to be monitored in just the axial direction (i.e., on one of the flat sides). For these samples, their weights were periodically monitored with a precision balance to calculate their Ca molar conversion to CaCO_3_. Additionally, the carbonate contents of certain samples extracted at different experimental times were checked by LECO analysis in order to estimate their maximum Ca molar conversion to CaCO_3_ (*X*_max_). To visually observe the carbonation mechanism, some porous samples were axially cut, and a phenolphthalein solution (1% wt in 96 %v ethanol) was sprayed on their interior surfaces. Phenolphthalein is a sensitive pH indicator that turns colorless in acid solutions and pink in basic solutions, thus allowing us to distinguish between Ca(OH)_2_ (colored) and CaCO_3_ (colorless).

Small samples (i.e., pellets 6 mm in diameter) and Ca(OH)_2_ parent solids (approximately 10–15 mg in powder form) were also tested in a TGA described elsewhere.^30^ The variations in sample weight over time under different temperatures (20 and 65 °C) and gas atmospheres (from compressed air with ppm_v_ CO_2_ up to 5 %v CO_2_ in air and different RHs up to 95% and a total gas velocity around the sample of about 0.02 m/s) were continuously monitored during these tests.

Finally, crushing strength (CS) tests of the dried samples were conducted and the evolution of CS with the carbonation degree was measured using a SHIMPO FGE-100X dynamometer. The peak force (in Newtons) required to break the samples^31^ was used as an indicator of their mechanical strength.

## Results and Discussion

**Figure 3:**
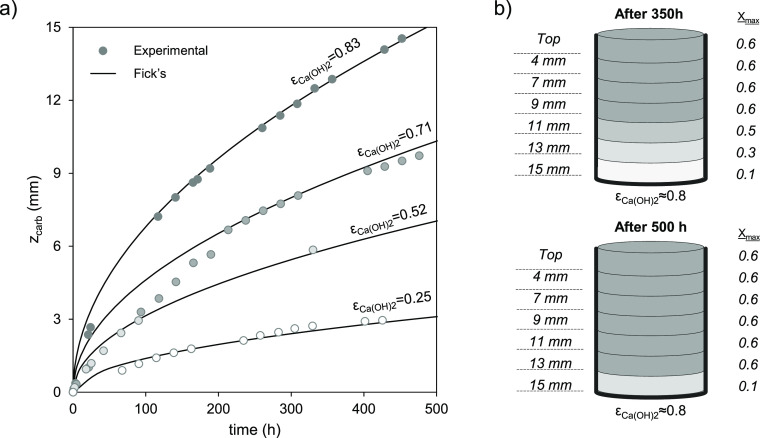
(a) Evolution of the depth of the carbonated layer (*z*_carb_) vs time during the carbonation of Ca(OH)_2_ samples with different porosities under testing room conditions (500 ± 25 ppm_v_ CO_2_, RH 52 ± 3% and 19 ± 2 °C). The lines are predictions from Fick’s law as in Eq 4 for samples with measured porosity ε_Ca(OH)_2__ of 0.83, 0.71, 0.52, and 0.25 and the best fit values of the tortuosity factor (τ of 1.20, 1.29, 1.30, and 1.85, respectively). (b) *X*_max_ obtained from LECO analyses at different approximate depths in samples with average ε_Ca(OH)_2__ ≈ 0.8 after 350 h (top) and 500 h (bottom).

The first set of carbonation experimental results obtained under the testing room ambient conditions (Figure 2 above) are presented in Figure 3, which was constructed by plotting the depth of the carbonated product layer, *z*_carb_, as a function of time up to 500 h. Such depth has been determined from the weight measurements of the samples, considering the established fact that the carbonation of Ca materials reaches maximum carbonation conversion *X*_max_ < 1 due to the formation of passivating CaCO_3_ product layer on the Ca(OH)_2_ surface^1,3,5^
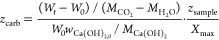
1

In Eq 1, *W*_0_ and *W*_*t*_ are the sample weights (in kg) measured at the beginning of the experiment and at different testing times, respectively; *M*_i_ is the molar mass of each compound (in kg/kmol); *w*_Ca(OH)2,0_ is the initial mass fraction of active Ca(OH)_2_ (considering CaCO_3_ as the main impurity in the powders); and *z*_sample_ is the axial height of the sample. The value of *X*_max_ is determined experimentally by analyzing the carbon contents of the different layers of carbonated material with a LECO analyzer. As shown in Figure 3b, all values of *X*_max_ reached a maximum stable value of 0.6 under this particular set of experimental conditions (a relative humidity of approximately 52% and a Ca(OH)_2_ surface area of 18.2 m^2^/g).

Figure 3a also includes lines that represent the predictions of a carbonation model considering the rate at which the carbonation front advances away from the exposed interface of the solid sample, with the ambient air being proportional to the flux of CO_2_ given by Fick’s law
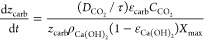
2where *D*_CO_2__ is the diffusion coefficient of CO_2_ in air (1.6 × 10^–5^ m^2^/s at 20 °C), ρ_Ca(OH)_2__ is the molar density of Ca(OH)_2_ (29.9 kmol/m^3^), τ is the tortuosity factor, C_CO2_ is the CO_2_ concentration (in kmol/m^3^), and *X*_max_ is the maximum carbonation conversion of the Ca(OH)_2_ material. Because the reaction is assumed to be controlled by the diffusion of CO_2_ in the stagnant volume of air contained in the porous carbonated layer resulting from the carbonation of Ca(OH)_2_, the porosity of this carbonated layer (ε_Carb_) is the one considered in Eq 2. This has been calculated assuming that there is no expansion of the porous solid during carbonation [i.e., ρ_Ca(OH)_2__·(1 – ε_Ca(OH)_2__) = ρ_CaCO_3__·(1 – ε_CaCO_3__), where ε_CaCO_3__ is the porosity of a CaCO_3_ layer and ρ_CaCO3_ is the molar density of CaCO_3_ of 27.1 kmol/m^3^] and considering the maximum carbonation conversion, as follows
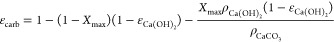
3

In an integrated form, Eq 2 gives the √*t* dependency similar to the one used in other studies of the slow carbonation of cementitious materials.^10,16,27,28^
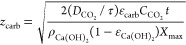
4

In Eqs 2–4, ε_Ca(OH)_2__ refers to the open porosity of the Ca(OH)_2_ samples. This value can be obtained by comparing the Ca(OH)_2_ true density with the one calculated by measuring the weight (*W*_0_) and total volume of the sample (*V*_sample_); thus, ε_Ca(OH)_2__ can be calculated as in Eq 5.
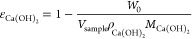
5

The best fits of Eq 4 to the experimental results noted in Figure 3a have been obtained when using tortuosity factor values of 1.20, 1.29, 1.30, and 1.85 for samples with ε_Ca(OH)_2__ of 0.83, 0.71, 0.52, and 0.25, respectively. These fitted τ values are consistent with those reported in the literature for other porous media.^32^

**Figure 4:**
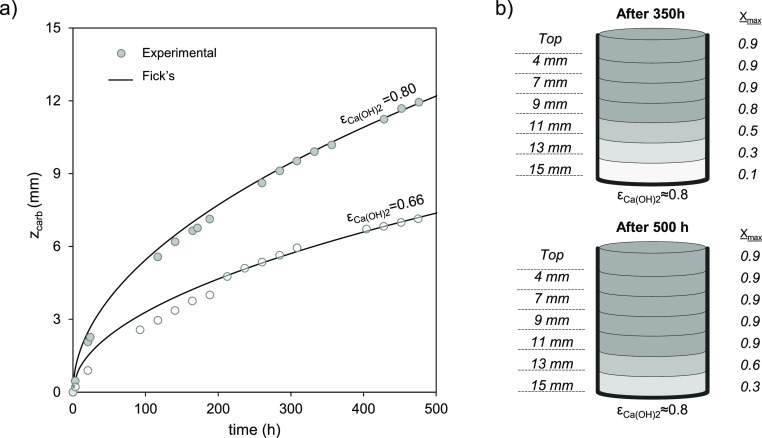
(a) Evolution of *z*_carb_ vs time during the carbonation experiments of compacted high-grade Ca(OH)_2_ powder with two different porosities under testing room conditions (500 ± 25 ppm_v_ CO_2_, RH 52 ± 3%, and 19 ± 2 °C). The lines are predictions from Fick’s law as in Eq 4 using τ values of 1.15 and 1.42 for ε_Ca(OH)_2__ of 0.80 and 0.66, respectively. (b) *X*_max_ obtained from LECO analyses at different depths for samples with average ε_Ca(OH)_2__ ≈ 0.8 after 350 h (top) and after 500 h (bottom).

Figure 4 shows similar experiments under testing room conditions but using samples of high-grade Ca(OH)_2_ (i.e., with a higher surface area of 39.3 m^2^/g vs 18.2 m^2^/g of those used in Figure 3) and ε_Ca(OH)_2__ values of 0.80 and 0.66 and fitted τ values of 1.15 and 1.42, respectively. The most remarkable difference is the much higher carbonation conversion of this material due to the increased specific surface, with *X*_max_ values reaching 0.85–0.90, as noted in Figure 4b. These results are consistent with those of previous studies on the carbonation of Ca(OH)_2_^1,3,5^ and can be explained by taking into account the impact on the maximum conversion of the passivating CaCO_3_ product layer. From a practical point of view, the use of these commercial high-surface-area Ca(OH)_2_ materials in direct air capture applications seems justified in view of both their larger CO_2_-capture capacity and maximum carbonation conversion and their lower requirements of relative humidity to achieve said maximum conversions.

The capability of Fick’s law (i.e., Eq 4) to properly fit the observations in Figures 3 and 4, once the values of *X*_max_, ε_Ca(OH)_2,__, and τ are considered, suggests that the carbonation reaction rates at the transition between the unreacted Ca(OH)_2_ and the layer of carbonated solids at *X*_max_ must be very fast compared to the rate of carbonation defined by Eq 4. In other words, it implies a rapid conversion, from 0 to *X*_max_, of the Ca(OH)_2_ grains near this reaction front. This is consistent with the results from studies of Ca(OH)_2_ carbonation,^1−3^ in which the high conversion of Ca(OH)_2_ to CO_2_ under suitable conditions of RH has been reported. As stated in the Introduction section, the importance of an intermediate H_2_O adsorption step in the mechanism of carbonation, enhanced by high values of RH, has been discussed in other works^1−5^ by proposing that the carbonation of Ca(OH)_2_ at temperatures below 100 °C takes place in solution, with the CO_2_ first dissolving in adsorbed layers of liquid water. Under low RH, the CaCO_3_ formed would cover the Ca(OH)_2_ surface more uniformly and passivate the material. In contrast, when RH increases (to values above 70%), the increased number of adsorbed water layers would provide a wider volume for the species to move all along the interfacial reaction regions and thus promote faster carbonation.^1,3,5^ Based on this, the proposed carbonation model of Eqs 2 and 4 has been applied only when the relative humidity, RH ≥ 50%.

**Figure 5:**
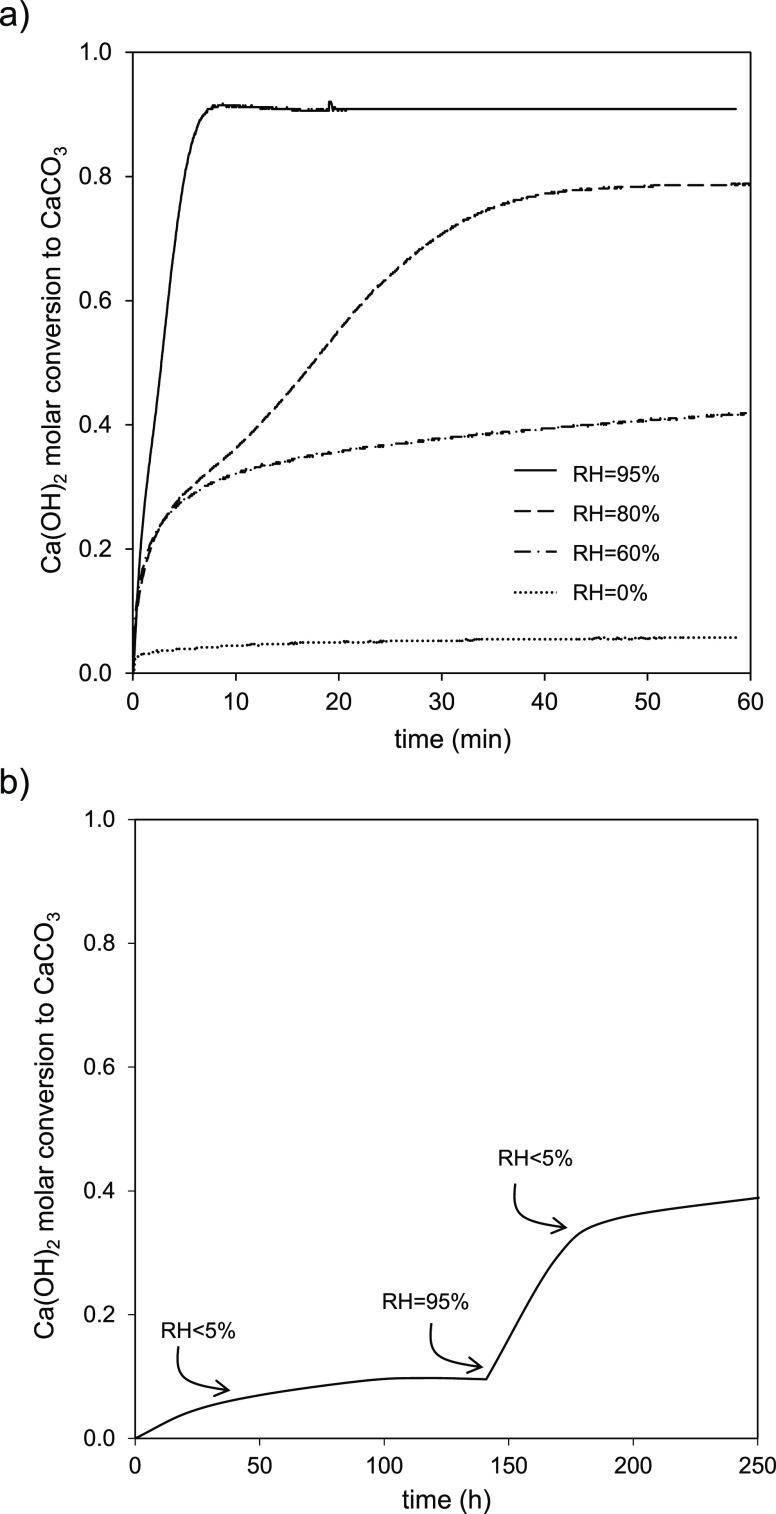
Ca(OH)_2_ molar conversion to CaCO_3_ vs time for different relative humidities (RHs) for high surface are Ca(OH)_2_ in the TGA: (a) powder at 65 °C and 5 %v CO_2_ and (b) pellet of 6 mm diameter carbonated under compressed air (450 ± 35 ppm_v_ CO_2_) at 20 °C.

To confirm the reported trends, we carried out dedicated experiments using TGA and intensified carbonation conditions, as shown in Figure 5. As seen, RHs close to saturation allowed a molar Ca conversion to CaCO_3_ over 0.9 in less than 10 min (see Figure 5a for the TGA of the carbonation at 65 °C and 5 %v CO_2_). After a first rapid step, the carbonation of Ca(OH)_2_ enters a slower phase. As the RH decreases, a transition in the carbonation rate appears at conversions between 0.2 and 0.3, with the rate of conversion progressively decreasing over time but still evolving slowly toward the maximum level of conversion reachable in time scales of several hours (not shown in the figure for simplicity). In contrast with the previous results, dry air with 5 %v CO_2_ (but RH = 0%) is unable to facilitate the carbonation of samples beyond conversions of 0.05.

This strong impact of RH on carbonation rates can also be observed in Figure 5b, although at different time scales. In this case, a Ca(OH)_2_ porous pellet is carbonated with air, and abrupt changes in the slope of the conversion curve were measured when changing from dry compressed air to fully saturated air. From a practical point of view, it is clear that RH values > 80% guarantee fast carbonation rates of any Ca(OH)_2_ material and the highest values of *X*_max_ at the grain level, making the overall carbonation process controlled by diffusion, as shown in Figure 5. Consistent with the proposed mechanism,^1−3,5^ and for long reaction times, it is also beneficial to use Ca(OH)_2_ materials with high surface areas when there is a need to moderate the RH values to just over 50% (e.g., to minimize the large consumption of water if a direct capture system is located in desert regions), as observed in the results reported in Figure 4. However, due to the extremely slow carbonation rate of Ca(OH)_2_ observed in dry air conditions,^1−4,6,9^ treating dry air should be avoided in future capture systems relying on the carbonation of Ca(OH)_2_.

**Figure 6:**
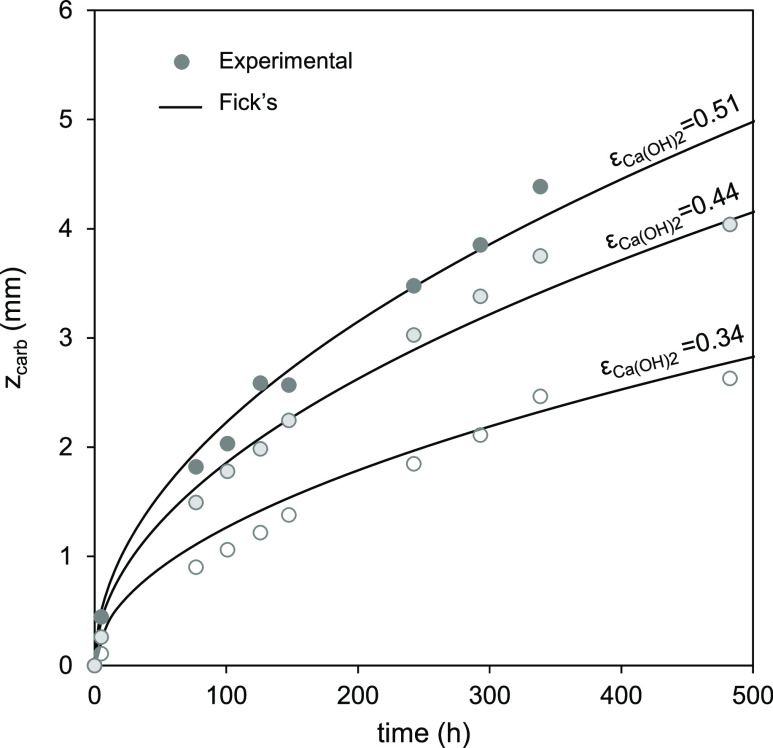
Evolution of *z*_carb_ vs time during the carbonation of extruded materials with different porosities tested in the sealed glass container fluxed with compressed air (450 ± 35 ppm_v_ CO_2_) saturated with humidity (i.e., RH > 95% and *X*_max_ approximately 0.9) at 20 °C. Lines represent the predictions from Fick’s law (Eq 4) for samples with ε_Ca(OH)_2__ of 0.51, 0.44, and 0.34 using τ values of 1.42, 1.49, and 1.95, respectively. Please note the different *Y*-axis scale with respect to other figures.

To further confirm these observations with extruded shapes at the macroscopic level, experiments were carried out in a sealed glass container fluxed with compressed air saturated with humidity (i.e., RH > 95%) on extruded materials of different porosities (i.e., with measured ε_Ca(OH)_2__ values of 0.51, 0.44, and 0.34). These were prepared using slaked Ca(OH)_2_ with a modest initial surface area. These tests revealed that RHs close to saturation enhanced the overall degree of carbonation of the sample by increasing the value of *X*_max_ to approximately 0.9 as measured by LECO analysis, making the use of high-surface-area Ca(OH)_2_ unnecessary. As shown in Figure 6, the curves of *z*_carb_ vs time can be well adjusted with Eq 4, as with previous samples, by fitting the τ values to 1.42, 1.49, and 1.95, respectively.

**Figure 7:**
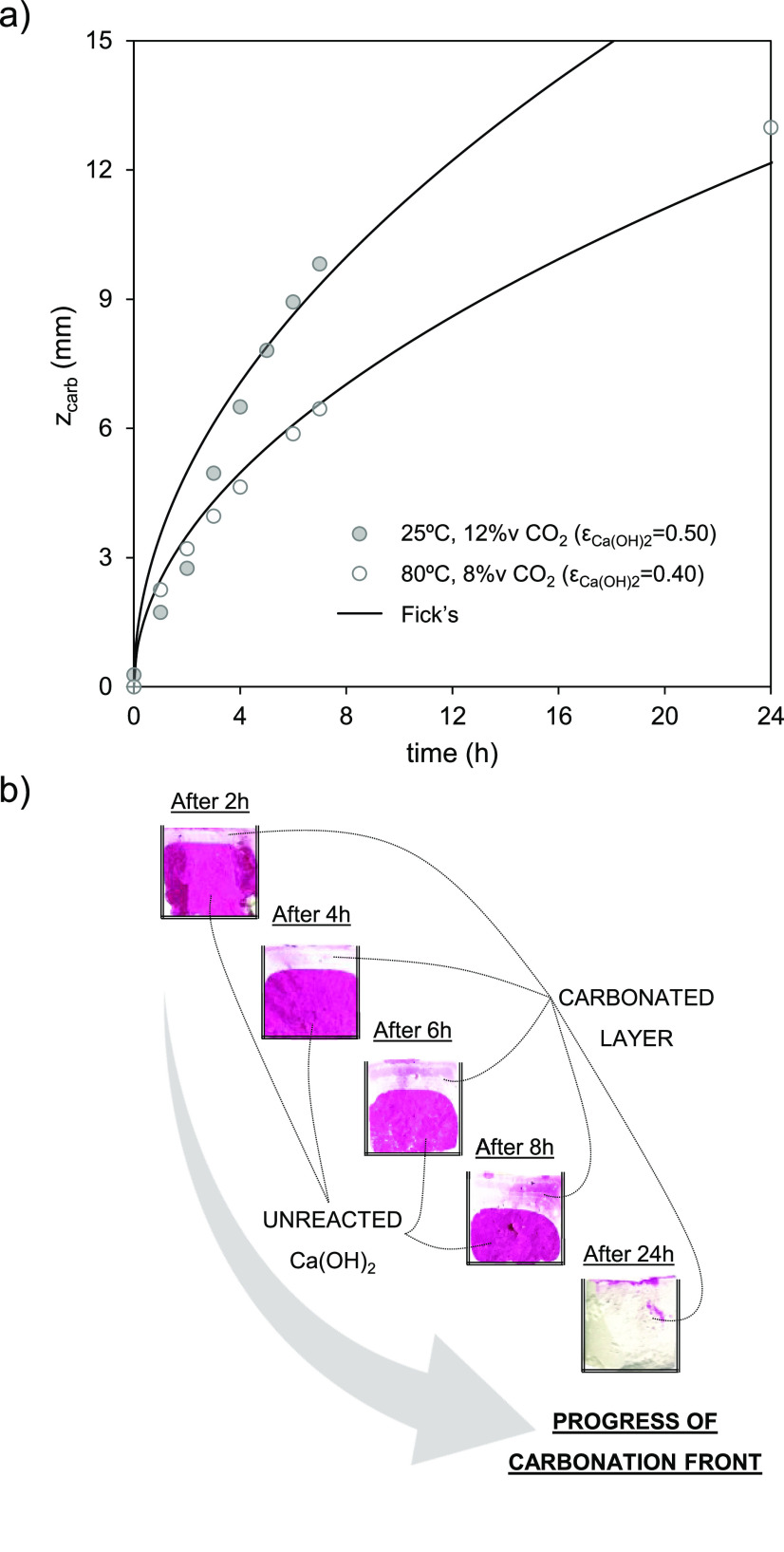
Evolution of *z*_carb_ vs time for dry mortar samples prepared from Ca(OH)_2_ powder tested in the sealed glass container at temperatures of 25 and 80 °C with air saturated with humidity (i.e., RH > 95%) and 12 %v and 8 %v CO_2_, respectively. (a) Experimental measurements and the predictions of Eq 4 using τ values of 1.42 and 1.55 for samples with ε_Ca(OH)_2__ of 0.50 and 0.40, respectively. (b) Images of samples extracted at different times and sprayed with a phenolphthalein solution over the axial section to follow the progress of the carbonation front.

**Figure 8:**
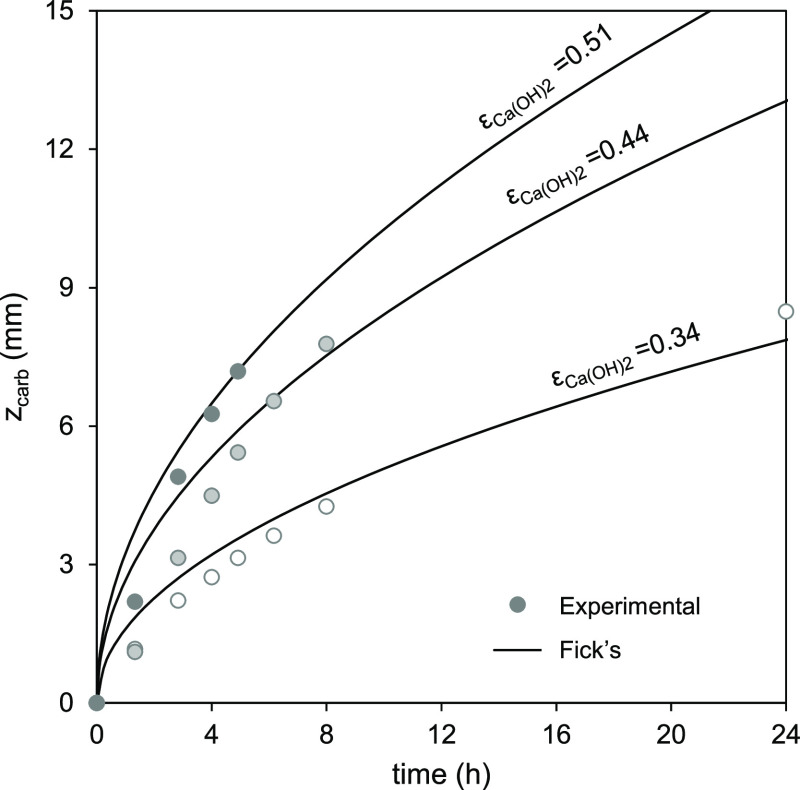
Evolution of *z*_carb_ vs time for extruded samples with different porosities tested in the sealed glass container at 25 °C with air saturated with humidity (i.e., RH > 95%) and 12 %v CO_2_ (dots) and the predictions of Eq 4 when τ is fitted to 1.62, 1.75, and 3.05 for samples with ε_Ca(OH)_2__ of 0.51, 0.44, and 0.34 (lines).

To assess the validity of Fick’s diffusion model under a wider range of conditions and time scales, accelerated tests using air with 2 orders of magnitude larger concentrations of CO_2_ (12 %v CO_2_ and 8 %v), characteristic of combustion flue gases, were carried out in sealed glass containers under humidity saturation conditions at 25 and 80 °C. Under these conditions, less than 24 h is sufficient to ensure the complete conversion of the samples, as shown in Figures 7a and 8 (for extruded materials, as in Figure 6). Remarkably, the fitting quality of Fick’s model in Figure 7a was retained for samples prepared from Ca(OH)_2_ powder with porosities of about 0.4–0.5 when using tortuosity factors comparable to those used in previous figures (i.e., τ of about 1.4–1.5). Figure 7b also shows the clear evolution of the depth of the carbonated layer over time by spraying a phenolphthalein solution over the axial section of the samples.

In the case of extruded materials of Figure 8, larger τ values are required to fit the experimental results using Eq 4 when compared to the non-extruded. Values of 1.62, 1.75, and 3.05 have been used to fit the experimental results obtained for samples with porosities of 0.51, 0.44, and 0.34, with the differences of special relevance in the case of the sample with the lowest porosity. This can be attributed to the existence of a certain fraction of inaccessible voids resulting from the higher pressures needed in the extrusion process to reach these lower porosities. Other effects, such as bottlenecks, could also reduce the effective porosity,^33^ thus increasing the tortuosity factor.

**Figure 9:**
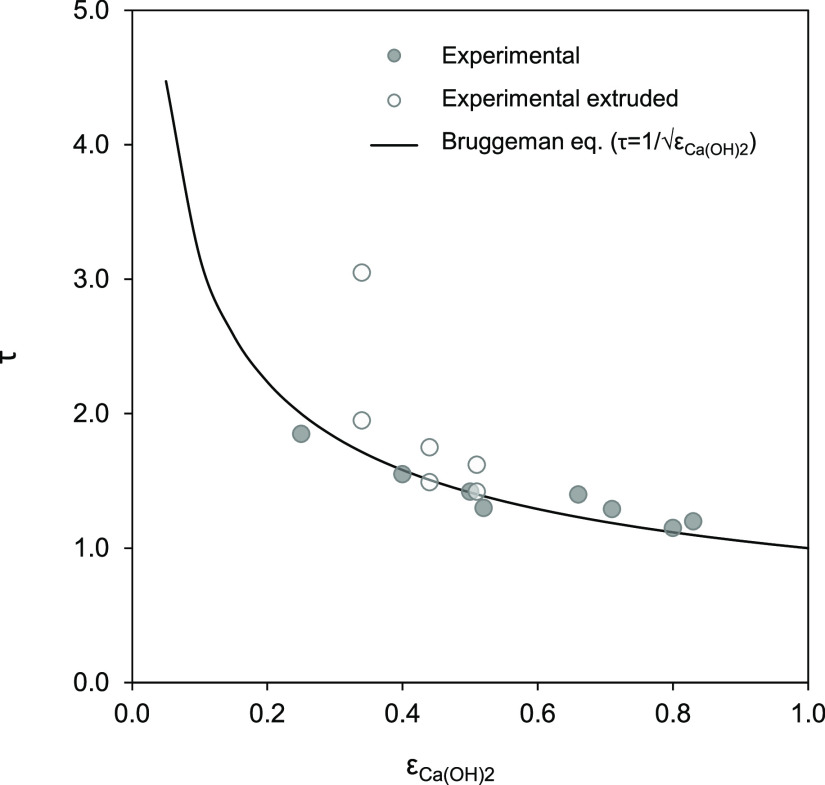
Tortuosity factor (τ) used in Eq 4 to fit the experimental results in Figures 3, 4, and 6–8 (dots) and calculated using the empirical Bruggeman equation^34^ (line) vs the open porosity of the samples (ε_Ca(OH)_2__).

In previous Figures 3, 4, and 6–8, the tortuosity factor in Eq 4 was used as an adjustable parameter to obtain the best fit of the Fick’s model to the experimental results. This parameter depends on porous media characteristics such as the porosity, pore diameter, channel shape, and so forth.^32^ In the absence of experimental data to fit such tortuosity factor, τ can be estimated from empirical equations such as that proposed by Bruggeman^34^ (relating tortuosity and porosity as τ = 1/√ε_Ca(OH)_2__). As shown in Figure 9, there is a good agreement between the fitted values from this work and those predicted by the empirical equation, with the main deviation observed in the extruded sample with the lowest porosity. As mentioned above, such discrepancy can be attributed to the formation of inaccessible voids during the extrusion process.

**Figure 10:**
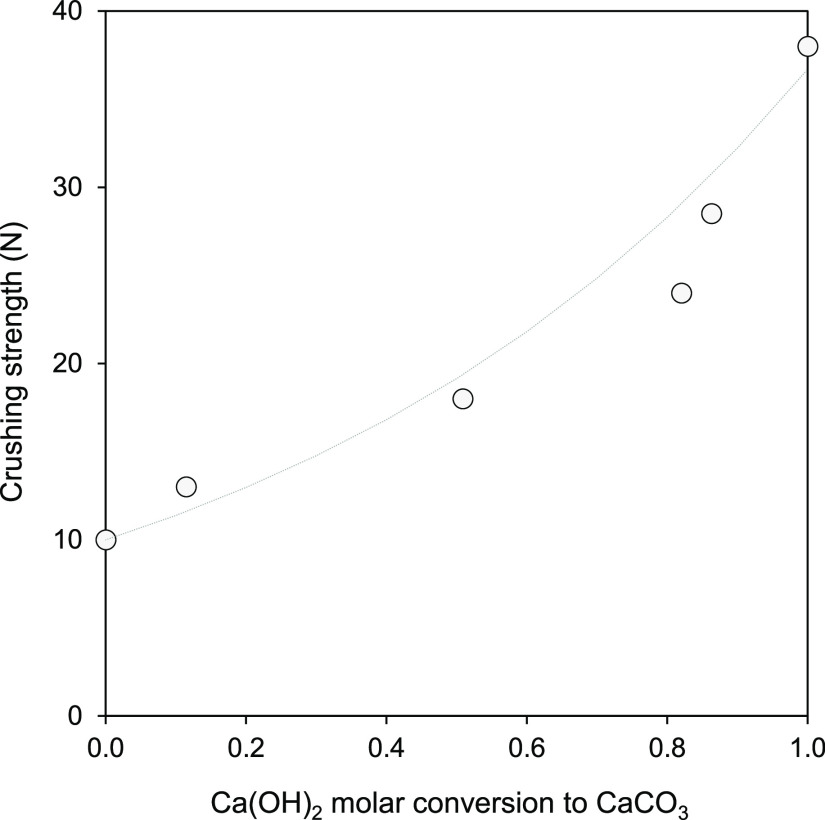
Evolution of the crushing strength (CS) with the Ca(OH)_2_ molar conversion to CaCO_3_ for 6 mm diameter pelletized samples tested in the sealed glass container under compressed air (450 ± 35 ppm_v_ CO_2_) saturated with humidity (i.e., RH > 95%) at 20 °C.

To complete the characterization of the carbonating samples, crushing strength measurements as a function of carbonation conversion were performed for some pelletized samples. As shown in Figure 10, the measured CSs significantly increased with the conversion of Ca(OH)_2_ to CaCO_3_. This improvement in CS with increasing levels of carbonation is consistent with observations of other Ca-based carbonating materials.^6,12,35−38^ Although it is beyond the scope of this work to elaborate on large-structure manufacturing aspects, the results of Figure 10 support the viability of future direct CO_2_-capture systems relying on the carbonation of large-scale Ca(OH)_2_ structures arranged to be exposed to air.^19,20^

## Conclusions

The carbonation of Ca(OH)_2_ porous solids on the millimeter-to-centimeter scale follows Fick’s diffusion law under a variety of CO_2_ concentrations in air (from 12 %v to ambient conditions at approximately 450 ppm_v_). The advance with time of the carbonation front for Ca(OH)_2_ samples in the form of pellets, dry mortars, and extrudes with porosities between 0.2 and 0.8 can be well fitted in most cases when considering the governing diffusion equation, the maximum carbonation conversion of the Ca solids, the porosities of the samples, and the tortuosity factor. The relative humidity (RH) in the air played an essential role in maximizing the Ca(OH)_2_ solid carbonation conversion, with an RH of >50% required to achieve maximum conversion values of 0.6. This maximum value of conversion can be increased by up to approximately 0.9 by using high-grade Ca(OH)_2_ (i.e., with specific surface areas of approximately 40 m^2^/g compared to the less than 20 m^2^/g measured for dry slaked limes) or RHs in the air close to saturation (i.e., RH > 95%). The crushing strengths of the carbonated Ca(OH)_2_ porous samples increased with increasing carbonate conversion, which supports the viability of the use of large structures composed of these solids to capture CO_2_ directly from air.

## Nomenclature

*C*_CO_2__: CO_2_ concentration, kmol/m^3^
*D*_CO_2__: diffusion coefficient of CO_2_ in air, m^2^/s
*M*_i_: molar mass of compound i, kg/kmol
*t*: time, s
*V*_sample_: total volume of the sample, m^3^
*W*_0_: initial sample weight, kg
*w*_Ca(OH)2,0_: mass fraction of active Ca(OH)_2_
*W*_*t*_: sample weight at time t, kg
*X*_max_: maximum Ca molar conversion to CaCO_3_
*z*_carb_: depth of the carbonated product layer, m
*z*_sample_: sample axial height, m
ε_Ca(OH)_2__: open porosity of the samples according to Eq 5
ε_carb_: porosity of the carbonated layer according to Eq 3
ρ_i_: molar density of compound i, kmol/m^3^
τ: tortuosity factor
